# Sexuality in Patients with Hidradenitis Suppurativa: Beliefs, Behaviors and Needs

**DOI:** 10.3390/ijerph17238808

**Published:** 2020-11-27

**Authors:** Carlos Cuenca-Barrales, Alejandro Molina-Leyva

**Affiliations:** 1 Hidradenitis Suppurativa Clinic, Dermatology Department, Hospital Universitario Virgen de las Nieves, 18014 Granada, Spain; carloscuenca1991@gmail.com; 2 TECe19-Clinical and Translational Dermatology Investigation Group, Instituto de Investigación Biosanitaria Granada, 18012 Granada, Spain; 3 European Hidradenitis Suppurativa Foundation (EHSF), 06847 Dessau-Roßlau, Germany

**Keywords:** hidradenitis suppurativa, cross-sectional studies, sexual health, sexual behavior, quality of life

## Abstract

Little is known about the impact of hidradenitis suppurativa (HS) on patients’ sexuality. The aim of this research is to investigate the impact of HS on several previously unexplored aspects of sexuality. In March 2018, we conducted a crowd-sourced cross-sectional online survey hosted by the Spanish association of patients with HS (ASENDHI) and available in Spanish. A panel of experts and patients from ASENDHI designed various questions in order to explore the extent to which HS influenced participants’ sex lives. The final sample consisted of 386 participants, 79.27% (306/386) of which were women and 20.73% (80/306) of which were men. Seventy-seven point one percent (236/306) of women and 67.5% (54/80) of men were in stable relationships; the rest of the participants were single. Forty-seven point nine percent (185/386) admitted to feeling fear of rejection. Pain was the symptom that most interfered with sexual relations in women and suppuration in men. Forty-four point three percent (171/386) of the participants considered themselves to be less attractive than average. Considering the participants in a stable relationship, women described receiving more emotional support from their partners, while men received more help with lesion dressing in intimate areas. Seventy-one point four percent (207/290) of participants stated that HS negatively affected their relationship. Among single patients, women experienced greater fear of rejection and were less willing to meet new people because of HS. Ninety-four point three percent (66/70) of women and 80.8% (21/26) of men stated that HS had a negative influence on their chances of having a relationship or sexual relations. In conclusion, HS has a significant, unrecognized and misunderstood impact on sexuality which must be addressed.

## 1. Introduction

Hidradenitis suppurativa (HS)/acne inversa is a recurrent, chronic, inflammatory, debilitating skin disease of the hair follicle that usually presents after puberty with painful, deep-seated, inflamed lesions in the apocrine gland-bearing areas of the body [1]. As the disease progresses, permanent scarring occurs in the form of sinus tracts. These lesions, besides pain, can cause suppuration, unpleasant odor and pruritus.

The World Health Organization (WHO) defines quality of life as an individual’s perception of their position in life in the context of the culture and value systems in which they live and in relation to their goals, expectations, standards and concerns [2]. It is a broad-ranging concept affected in a complex way by the person’s physical health, psychological state, personal beliefs, social relationships and their relationship to salient features of their environment [2]. Recent studies show that the reduction in quality of life in patients with HS is one of the most significant among dermatological patients and comparable to other diseases such as cancer, diabetes, cardiovascular disease and chronic obstructive pulmonary disease [3,4,5].

According to WHO, sexual health is a state of physical, mental and social wellbeing in relation to sexuality [6]. Sexuality is considered to be quite important for maintaining good mental health and a basic need that cannot be separated from other aspects of human life [7]. Several studies indicate that sexual function is directly related to quality of life [8,9]. Chronic diseases can have a negative impact on sex life through different mechanisms [10].

Psoriasis is the most investigated chronic dermatological disease in terms of its impact on patients’ sexuality. Measured using validated questionnaires, the prevalence of sexual dysfunction varies between research from 40% to 55.6%, with patients with psoriasis having a risk of sexual dysfunction 5.5-fold higher than healthy controls [11]. The presence of anxiety or depression, female sex, increasing age, psoriatic arthritis, genital psoriasis and psoriasis severity are factors possibly associated with sexual dysfunction [11]. Several biologic drugs seem to improve sexual dysfunction in psoriasis patients [11].

In a multicentric study in 13 European countries, 23.1% of 3485 patients reported sexual difficulties, with hidradenitis suppurativa, prurigo, blistering disorders, psoriasis, urticaria, eczema and skin infections being the diseases with highest impairment [12]. This impact was associated with anxiety, depression and suicidal ideation [12]. However, the research did not use validated questionnaires to explore sexual dysfunction, but instead used question 9 of the Dermatology Life Quality Index.

Regarding HS, the prevalence of sexual dysfunction in women measured using validated tools ranges from 51% to 62%, and that of erectile dysfunction in men from 52% to 60% [13]. Factors associated with sexual dysfunction in women with HS are higher educational status, disease activity, intensity of pain and unpleasant odor, absence of a stable relationship and older age at HS onset, while in men, the factors are ageing, active lesions on the genitals and increased number of active lesions [13]. Patients with HS also suffer from more sexual distress than the healthy population; factors related to sexual distress are female sex, active lesions in the groin and on the genitals, intensity of unpleasant odor and pain and the absence of a stable relationship [13]. Sexual function is correlated with quality of life and with mood status disturbances (anxiety and depression) [13]. However, there is no information about how HS hinders sex life, how patients perceive themselves, whether they experience interference in their relationships or opportunities to find a partner and, if so, what this interference is. We hypothesize that HS has a high impact on these unexplored aspects of sexuality, and the aim of this study is to investigate this and to answer the following questions: Which difficulties do patients with HS experience in sexual activity? Would they like to share these difficulties with healthcare staff? What is their sexual orientation? How attractive do they feel? How does HS influence patients who are in relationships? How does HS influence patients who are single?

## 2. Materials and Methods

### 2.1. Patients and Methods

We conducted a cross-sectional study by means of a crowd-sourced online survey from 1 March to 1 April 2018. The Spanish hidradenitis suppurativa patients’ association (ASENDHI) hosted the survey on its website and posted the survey on its social media networks [14].

The selection criterion was self-referred diagnosis of HS. The study was approved by the Institutional Review Board of Hospital Universitario San Cecilio (Granada, Spain, IRB code 0105-N-20) and is in accordance with the Declaration of Helsinki. Participants were informed about the survey’s anonymity and the use of their data for research purposes, and they received no compensation for participating in the survey.

### 2.2. Questionnaire

The questionnaire was developed with Google Forms^®^ suite and was provided in Spanish. The data presented belong to a research project about sexuality and HS. Results regarding sexual and erectile dysfunction, sexual distress and associated factors have been already published elsewhere [15,16].

In this study, questions were created both by a panel of experts and a group of patients from ASENDHI in order to evaluate the extent to which the disease has an influence on participants’ sex lives. The survey included questions about difficulties in their sex life due to HS, which healthcare professional they would trust to share their sexual problems with, perceived attractiveness and sexual orientation. In the case of patients in a stable relationship, there were also questions about the role of their partners and how HS affects their relationships. In the case of single patients, there were also questions about their feelings when they meet new people and how HS affects their opportunities to meet people/establish a relationship. We did not use validated questionnaires because there are no tools available to explore these important factors related to sexuality and private life, since other questionnaires merely explore the presence or absence of sexual dysfunction and, as mentioned above, they have already been used on HS [15,17,18,19].

Since the questions used were new and not validated, a control group of healthy acquaintances was also recruited by ASENDHI and a survey with all the questions not directly related to HS was distributed among them. The questions used in the present manuscript for both patients with HS and healthy controls are available as supplementary material in the original Spanish version and in an English translation (Supplementary Material File S1).

Sociodemographic data, biometric parameters, use of medication for other comorbidities and several characteristics of the disease were also collected. Disease severity was assessed by patients’ self-reported Hurley stage since patients with HS are capable of self-assessing their Hurley stage with a good correlation with physician assessment [20].

Disease activity was assessed by the Patients’ Global Assessment (PtGA) scale, consisting of five categories (inactive, very low, low, mild and severe) [21], and intensity of symptoms by Numeric Rating Scales (NRS) [22], where 0 is the lowest and 10 the highest intensity of the symptom. These scales show the subjective impact of the disease on patients, with equal or greater importance than objective scales [23,24].

### 2.3. Statistical Analyses

Statistical analyses were performed using the IBM software Statistical Package for Social Science version 23.0 (SPSS Inc, Chicago, IL, USA). When data were missing from any of the variables of interest, patients were excluded from the study. When missing data were found in other variables, they were imputed. We used the Shapiro–Wilk test and histograms to assess if the variables were normally distributed. Descriptive statistics were used to explore the characteristics of the sample. Continuous variables were expressed as means and standard deviations (SD) or as medians and interquartile ranges (IR). Qualitative variables were expressed as absolute and relative frequencies.

We explored factors associated with fear of rejection/reaction of the sexual partner, perceived attractiveness, interference in relationships and interference in meeting new people by means of multivariate logistic regressions, including as variables age, sex, body mass index (BMI), educational status, age at HS onset, PtGA, NRS for pain, suppuration and unpleasant odor, self-reported Hurley, the number of areas affected by active lesions, the number of areas affected by scars, the presence of active lesions in the groin and genitals and, in the case of fear of rejection/the reaction of the sexual partner and perceived attractiveness, the presence of a stable relationship. Perceived attractiveness, interference in relationships and interference in meeting new people were binary codified (yes/no) for this analysis. Significance was set for all tests at two tails, *p* < 0.05.

## 3. Results

### 3.1. Baseline Characteristics

Three hundred and ninety-three participants with HS completed the questionnaire, seven of them incompletely. The final sample therefore consisted of 386 participants, 79.27% (306/386) of which were women and 20.73% (80/386) men, resulting in a male–female ratio of 3.8:1. Their mean age was 37.81 (9.26) years old. Seventeen point six percent (68/386) of the patients were in Hurley stage I, 45.1% (174/386) in stage II and 37.3% (144/386) in stage III (Table 1). We also collected 157 healthy controls, with no statistically significant differences from patients with HS in terms of sex (73.25% (115/157) were women and 26.75% (42/157) men, *p* = 0.16) or age (mean age = 37.5 (12.03) years old, *p* = 0.74). BMI was significantly lower in healthy controls (24.66 (4.47) kg, *p* < 0.001).

When comparing patients with HS with and without a stable partner, there were no differences in genital involvement (35.5% (103/290) vs. 40.6% (39/96), respectively, *p* = 0.37 for the difference), inguinal involvement (64.1% (186/290) vs. 60.4% (58/96), *p* = 0.51), PtGA (3.7 vs. 3.6, *p* = 0.62), number of active regions (2.5 (0.1) vs. 2.7 (0.2), *p* = 0.4), NRS for pain (6.7 (0.2) vs. 6.2 (0.3), *p* = 0.18) or Hurley stage (I: 16.2% (47/290), II: 44.1% (128/290), III: 39.7% (115/290) vs. I: 21.9% (21/96), II: 47.9% (46/96), III: 30.2% (29/96), *p* = 0.2). NRS for unpleasant odor was higher among patients with a stable partner (5.9 (0.2) vs. 4.8 (0.3), *p* < 0.01).

### 3.2. Perceived Difficulties in Sexual Activity

When participants were asked how HS affected their sexual relationships (Table 2), almost half reported feeling fear of rejection or of the reaction of their sexual partner, while in the healthy control group, 37.6% (59/157) reported feeling fear of rejection at some point in their lives (*p* < 0.05), mainly due to insecurity (27.39%, 43/157) and concerns about physical appearance (26.12%, 41/157). Factors significantly associated with this feeling in patients with HS were younger age (OR = 1.05 (95% CI: 1.02–1.08), *p* < 0.001), the absence of a stable relationship (OR = 7.72 (4.24–14.06), *p* < 0.0001) and the number of areas affected by scars (OR = 1.14 (1.02–1.28), *p* < 0.05). We found trends toward statistical significance in self-reported Hurley (III vs. I, OR = 2.06 (0.97–4.38), *p* = 0.059). Patients also felt that symptoms significantly affected their relationships, with pain being the most important symptom in women (65.4%, 200/306) and suppuration in men (56.3%, 45/80). Some participants also thought that treatment made sexual relations difficult (10.6%, 41/386).

Regarding the healthcare staff they would share their sexual problems with, a considerable percentage of participants with HS would not talk with anyone (37.6%, 145/386); women preferred a psychologist or a sexologist (41.2%, 126/306), and men felt most comfortable with their dermatologist (31.3%, 25/80) (Table 2). This is the opposite to the healthy control group, where most would like to share their sexual problems (95.5%, 150/157, *p* < 0.0001), mainly with a psychologist or a sexologist (82.2%, 129/157).

### 3.3. Sexual Orientation

Regarding sexual orientation, most participants were heterosexual (87.5% (70/80) of men and 94.8% (290/306) of women). Eleven point three percent (9/80) of men and 1.3% (4/306) of women were homosexual and 1.3% (1/80) of men and 3.9% (12/306) of women were bisexual. Sexual orientation was similar in the control group: 88.1% (37/42) of men and 89.57% (103/115) of women were heterosexual, 11.9% (5/42) of men and 3.48% (4/115) of women were homosexual, and 6.95% (8/115) of women were bisexual.

### 3.4. Perceived Attractiveness

Participants were also asked to rate their perceived sexual attractiveness (Table 3). The differences with the control group were again significant (*p* < 0.0001). It is noteworthy that 38.75% (31/80) of men and 45.75% (140/306) of women with HS considered themselves as “less attractive than average” or “not at all attractive”. Factors related to lower perceived attractiveness in patients with HS were NRS for unpleasant odor (OR = 1.14 (1.04–1.26), *p* < 0.01), NRS for suppuration (OR = 1.14 (1.01–1.27), *p* < 0.05) and the absence of a stable relationship (OR = 1.81 (1.1–2.96), *p* < 0.05); the presence of active lesions in the groin showed trends toward statistical significance (OR = 1.62 (0.97–2.7), *p* = 0.07).

### 3.5. Sexuality in Patients with HS in a Stable Realtionship

Sixty-seven point five percent (54/80) of men and 77.1% (236/306) of women were in a stable relationship. Although it was not statistically significant, women reported more support (*p* = 0.22) and greater help with overcoming the fear of rejection (*p* = 0.09) than men, whereas help with lesion dressing in intimate areas was higher in men (*p* < 0.05).

It is significant that around 70% of participants, regardless of sex, state that HS negatively affects their relationship (Table 4). PtGA was related to the perception of relationships being negatively affected (OR = 1.45 (1.01–2.08), *p* < 0.05); the number of areas affected by active lesions showed trends toward statistical significance (OR = 1.3 (0.98–1.72), *p* = 0.06).

### 3.6. Sexuality in Single Patients with HS

Thirty-two point five percent (26/80) of men and 22.9% (70/306) of women were single. When patients were asked how they felt when they meet someone they could have a relationship or sexual relations with, women experienced greater fear of rejection (*p* = 0.11) than men, and the percentage of women who did not want to meet people because of HS was also higher (*p* = 0.22). Less than a quarter of participants felt excited when they met new people. On the other hand, in the control group, 77.5% (31/40) of the participants felt excited when meeting new people (*p* < 0.0001) and 22.5% (9/40) stated fear of rejection of the new partner for whatever reason (*p* < 0.0001).

A striking fact is that almost 95% of women and more than 80% of men stated that HS had a negative influence on their chances of having a relationship or sexual relations (Table 5). We did not find any significant association of the explored factors with this feeling.

## 4. Discussion

In this study, several previously unexplored factors related to sexuality in patients with HS have been assessed. Previous research has shown that sexual and erectile dysfunction have a high prevalence in patients with HS, that they suffer greatly from sexual distress and that this is related to a lower quality of life [13]. In this investigation, we have seen that HS has an important, unrecognized and misunderstood impact on sexuality which must be addressed. Most patients experience feelings of fear in their relationships, and factors such as symptoms or the absence of a stable partner play an important role in this impact on sexuality. However, both single patients and those in a stable relationship are affected.

### 4.1. Baseline Characteristics

In general terms, the baseline characteristics of the sample, such as disease or sociodemographic features, including the male–female ratio, were similar to those reported in other studies and representative of the general HS population [25,26,27,28,29,30,31,32]. As expected, BMI was significantly higher in patients with HS than in healthy controls, given the association of this disease with being overweight and obesity [33]. However, BMI was considered in multivariate analyses, and no association was found with any of the explored aspects of sexuality.

### 4.2. Perceived Difficulties in Sexual Activity

Symptoms seem to play an important role in the sexual impairment of patients with HS. Women perceived pain as the symptom which most affected their sexual relations. This is consistent with several studies indicating that women have a greater perception of pain and an increased risk of developing chronic pain, based on various mechanisms such as endogenous opioid activity, sex hormones, genetic factors and psychosocial factors [34,35,36]. On the other hand, suppuration was identified by men as the symptom which most affected their sexual relations. The interference of suppuration in sexual relations is probably due to factors related to the nature of the sexual act and/or to psychological factors that could be associated with disease activity [3] and probably also the reason for the observed association between the NRS for suppuration and unpleasant odor with a lower perceived attractiveness.

Several treatments for HS can affect patients’ sex lives, such as surgical procedures, topical ointments and lesion dressings. We found a lower percentage of sexual impairment due to HS treatment than Janse et al. [18], maybe because their sample came in part from a hospital setting.

It should be noted that almost 1 in 3 participants would share their sexual problems with their dermatologist, and almost 2 in 5 with a psychologist or sexologist. According to Janse et al. [18], only 6% of patients with HS reported receiving enough attention from their doctor about their sexual problems, probably due to the complexity of sexual problems, embarrassment about tackling the issue, difficulty of treatment and lack of time. Given the profound impact of HS on sexuality, it would be advisable to routinely screen these patients for sexual problems, offering them follow-up attention in a specialized unit if they so require. Moreover, it is necessary to create an atmosphere of trust with these patients, since 1/3 do not want to talk about their sexual problems, in contrast to more than 95% of the healthy controls, who would discuss their sexual problems. It seems that patients are so embarrassed by their disease that they prefer not to share their problems, so it is important to create a relationship which allows them to freely express their concerns.

### 4.3. Sexual Orientation

As far as we know, this is the first study evaluating the sexual orientation of patients with HS. We did not observe any differences compared to the healthy controls, as has been observed in previous research on patients with psoriasis [37,38].

### 4.4. Perceived Attractiveness

More than 2/5 of participants considered themselves less attractive than average, and almost half reported being afraid of rejection and of the reaction of their sentimental/sexual partner, probably due to the impact of HS on self-body image [39], as also occurs in psoriasis [40]. These results were significantly higher than those found in healthy people. Younger patients were at greater risk of experiencing these feelings, probably because they had not yet developed coping mechanisms. The relation observed between fear of rejection and the number of areas affected by scars, as well as the trend towards significance for self-reported Hurley, is in concordance with this impact on self-body image. Like patients with burns, body image disturbances may be due to dysfunctional coping strategies, and appropriate intervention in this area could lead to an improvement in these feelings [41]. These alterations are associated with sexual impairment due to inhibition and shyness, generating less excitability and less ability to achieve orgasm, regardless of sex and whether or not in a stable relationship [42]. However, we observed that the absence of a stable relationship was related to greater fear of rejection and to lower perceived attractiveness, probably because the trust generated in a relationship could alleviate the negative feelings previously described, with less self-consciousness and less orgasm difficulty in both men and women [42].

### 4.5. Sexuality in Patients with HS in a Stable Relationship

Even when body image alterations have a lower impact on sexual health in people with a stable partner [42], they can still influence the relationship quality as perceived by the subject [43]. Around 70% of patients in a stable relationship referred to support from their partners and around half help them to overcome fear of rejection and help with lesion dressing in intimate areas. In addition, around 70% of the patients stated that the disease has little or no influence on their relationships, reflecting the importance of partner support in the feelings experienced by the patients. However, around 1/3 thought that HS had a significant negative influence on their relationship. These data agree with previous studies showing lower quality of life in the cohabitants of patients with psoriasis [44] and HS [45,46]. Another investigation observed a higher prevalence of intimate partner violence, but no increased risk of sexual assault, in patients with HS than in patients with acne, indicating a possible deterioration of life as a couple [47]. Therefore, the disease seems to have a significant impact on relationships. Disease activity was related to this impact and the number of areas affected by active lesions showed trends towards statistical significance, so proper individualized treatment could improve patients’ relationships.

### 4.6. Sexuality in Single Patients with HS

We observed that patients with factors related to sexual dysfunction or sexual distress in HS (genital and inguinal involvement, higher PtGA, higher number of affected areas or higher NRS for pain and for unpleasant odor) [15,16], as well as those with a higher Hurley stage, were not more likely to be single. The percentages of single women who felt fear of rejection when meeting a possible sexual partner and of people who preferred not to meet anyone because of the disease were higher than single men with HS, although not statistically significant. On the other hand, the differences between patients and healthy controls in terms of feelings of excitement or fear of rejection when meeting new people were significant. More than 90% of participants thought that HS influenced their ability to find a stable/sporadic partner, and more than half reported that it influenced this ability “somewhat” or “very much”, this percentage again being higher in women. These data are worrying and reflect the profound impact of the disease on sexuality and patients’ confidence. Several studies indicate that young adults with chronic illnesses are less likely to get married [48,49,50]. It seems that people with chronic diseases have less self-confidence, decreasing their chances of finding a partner and thus getting into a vicious cycle of sexual distress.

### 4.7. Limitations

Our investigation has some limitations. Firstly, there is a possible selection bias, since it only represents patients in contact with support groups and with internet access. Elderly patients who might use the internet less frequently, or those with low socio-cultural status or fear of new technologies, might be under-represented [51]. However, the baseline characteristics of our sample do not differ significantly from those reported in other research about HS, either in hospital-based or population-based studies. Secondly, there was a possible classification bias, since it was an online questionnaire so we could not confirm the HS diagnosis and HS characteristics, which were self-reported. Nevertheless, an informed population can properly identify HS due to its apparent and distinctive clinical manifestations. Given the questionnaire’s dissemination through a patients’ association, it is expected that the participants did in fact suffer from the disease. Finally, the questions used in this study are not validated; however, as discussed above, there are no validated tools available to assess the aspects of sexuality explored in this research, and we have compared many of the questions (all those which did not ask directly about the disease) with a healthy control group.

## 5. Conclusions

In conclusion, HS has a significant impact on important aspects of quality of life, such as sexuality and private life. Symptoms, and sometimes treatment, affect sexual activity. Many patients experience greater feelings of rejection and lower perceived attractiveness than healthy people. Body image alteration may play an important role, as it demonstrates the association between fear of rejection and the number of areas affected by scars. The absence of a stable relationship is also an important risk factor. However, there is also a noticeable impact on stable relationships, in part due to disease activity. A high percentage of single participants thought that the disease decreased their possibilities of having a sexual/sentimental partner, reflecting their sexual distress and lack of confidence. A considerable number of participants would like to share their sexual problems with health professionals, so patients should be asked about their sexual difficulties and specialized attention should be provided when necessary.

## Figures and Tables

**Table 1:** Sociodemographic characteristics, comorbidities and baseline characteristics of patients with HS and healthy controls.

Baseline Characteristics	Men (*n* = 80)	Women (*n* = 306)	All (*n* = 386)	Controls (*n* = 157)	*p* Value *
**Age** (years)	39.21 (11.15)	37.44 (8.69)	37.81 (9.26)	37.05 (12.03)	0.74
**Residence country**					0.13
Spain	90% (72)	80.7% (247)	82.6% (319)	89.2% (140)	
Other **	8.7% (7)	19.3% (50)	14.8% (57)	10.2% (16)	
Not answered	1.3% (1)	2.9% (9)	2.6% (10)	0.6% (1)	
**Educational status**					0.14
Basic	16.3% (13)	11.8% (36)	12.7% (49)	8.3% (13)	
Medium	32.5% (26)	35.6% (109)	35% (135)	31.8% (50)	
Superior	51.2% (41)	52.6% (161)	52.3% (202)	59.9% (94)	
**BMI** (kg)	28.12 (5.03)	29.67 (7.05)	29.35 (6.71)	24.66 (4.47)	**<0.001**
**Current smoker**					0.15
No	35% (28)	44.1% (135)	42.2% (163)	47.1% (74)	
Yes	65% (52)	55.9% (171)	57.8% (223)	52.9% (83)	
**Comorbidities**					0.11
HBP	5% (4)	6.9% (21)	6.5% (25)	4.5% (7)	
DM2	2.5% (2)	6.5% (20)	5.7% (22)	3.1% (5)	
Dyslipidemia	3.8% (3)	2.9% (9)	3.1% (12)	1.3% (2)	
IBD	1.3% (1)	0.7% (2)	0.8% (3)	0% (0)	
Antidepressant use	5% (4)	10.1% (31)	9.1% (35)	5.7% (9)	
Benzodiazepine use	5% (4)	5.9% (18)	5.7% (22)	5.1% (8)	
Levothyroxine use	-	7.8% (24)	6.2% (24)	4.5% (7)	
Hyperuricemia	3.8% (3)	0.7% (2)	1.3% (5)	0.5% (1)	
Asthma/Seasonal allergies	-	1.6% (5)	1.3% (5)	2.6% (4)	
**Stable relationship**	67.5% (54)	77.1% (236)	75.1% (290)	74.5% (117)	0.88
**Age of onset** (years)	23.57 (9.45)	19.09 (7.1)	20.02 (7.85)		
**Time of disease evolution** (years)	15.64 (10.53)	18.33 (9.3)	17.77 (9.62)		
**Time under medical attention** (years)	6.79 (7.21)	7.1 (7.29)	7.03 (7.27)		
**Diagnosis delay** (years)	8.86 (9.13)	11.23 (9.55)	10.74 (9.51)		
**Locations**					
**Axilla**					
Active lesions	50% (40)	47.1% (144)	47.7% (184)		
Scars	47.5% (38)	43.8% (134)	44.6% (172)		
**Groin**					
Active lesions	53.8% (43)	65.7% (201)	63.2% (244)		
Scars	42.5% (34)	57.2% (175)	54.1% (209)		
**Genitals**					
Active lesions	38.8% (31)	36.3% (111)	36.8% (142)		
Scars	35% (28)	26.8% (82)	28.5% (110)		
**Buttocks**					
Active lesions	43.8% (35)	31% (95)	33.7% (130)		
Scars	35% (28)	32.4% (99)	32.9% (127)		
**Breast**					
Active lesions	2.5% (2)	29.4% (90)	23.8% (92)		
Scars	6.3% (5)	27.1% (83)	22.8% (88)		
**Abdomen**					
Active lesions	11.3% (9)	10.1% (31)	10.4% (40)		
Scars	10% (8)	11.8% (36)	11.4% (44)		
**Perianal region**					
Active lesions	43.8% (35)	16.3% (50)	22% (85)		
Scars	31.3% (25)	18% (55)	20.7% (80)		
**Neck**					
Active lesions	13.8% (11)	4.6% (14)	6.5% (25)		
Scars	11.3% (9)	4.6% (14)	6% (23)		
**N° of active regions**	2.73 (1.79)	2.5 (1.57)	2.55 (1.62)		
**N° of regions with scars**	2.34 (2.29)	2.31 (2.06)	2.31 (2.1)		
**Hurley stage**					
I	16.3% (13)	18% (55)	17.6% (68)		
II	31.3% (25)	48.7% (149)	45.1% (174)		
III	52.5% (42)	33.3% (102)	37.3% (144)		
**PtGA**	3.73 (1.04)	3.65 (1.11)	3.66 (1.09)		
**NRS pain**	6.64 (2.81)	6.52 (2.98)	6.54 (2.95)		
**NRS pruritus**	6.24 (2.67)	6.48 (3.03)	6.43 (2.96)		
**NRS unpleasant odor**	6.11 (3.05)	5.47 (3.45)	5.6 (3.38)		
**NRS suppuration**	6.84 (3.04)	6.39 (3.21)	6.48 (3.18)		

Continuous variables are expressed as means (standard deviation) and qualitative variables as relative (absolute) frequencies. HS: hidradenitis suppurativa. BMI: body mass index. HBP: high blood pressure. DM2: diabetes mellitus type 2. IBD: inflammatory bowel disease. PtGA: Patient’s Global Assessment; values range from 1 (inactive disease) to 5 (severe disease). NRS: Numeric Rating Scale; values range from 0 (no symptoms) to 10 (maximum intensity of symptoms). * *p* value for the difference between HS patients and healthy controls. Significant values (*p* < 0.05) are in bold. ** Other includes Germany, Argentina, Australia, Chile, Colombia, Costa Rica, Ecuador, USA, Italy, Mexico, Peru, Portugal, Switzerland, Uruguay and Venezuela.

**Table 2:** Perceived difficulties in sexual relations in patients with HS.

Perceived Difficulties in Sexual Relations	Men (*n* = 80)	Women (*n* = 306)	All (*n* = 386)
**How does HS affect your sexual relations?**			
“I experience fear of rejection or of the reaction of my sexual partner”	47.5% (38)	48% (147)	47.9% (185)
“Pain interferes with my sexual relations”	48.8% (39)	65.4% (200)	61.9% (239)
“Suppuration interferes with my sexual relations”	56.3% (45)	43.5% (133)	46.1% (178)
“Odor interferes with my sexual relations”	40% (32)	31.4% (96)	33.2% (128)
“Treatment interferes with my sexual relations”	13.8% (11)	9.8% (30)	10.6% (41)
“None of the above”	18.8% (15)	9.5% (29)	11.4% (44)
**Would you like to share your sexual problems with healthcare staff?**			
“No”	46.3% (37)	35.3% (108)	37.6% (145)
“Yes, with my GP”	8.8% (7)	15.7% (48)	14.2% (55)
“Yes, with my dermatologist”	31.3% (25)	26.8% (82)	27.7% (107)
“Yes, with nursing staff”	2.5% (2)	5.6% (17)	4.9% (19)
“Yes, with a psychologist/sexologist”	27.5% (22)	41.2% (126)	38.3% (148)

Results are expressed as relative (absolute) frequencies. HS: hidradenitis suppurativa. GP: general practitioner.

**Table 3:** Perceived attractiveness in patients with HS and healthy controls.

Perceived Attractiveness	Patients with HS (*n* = 386)		Healthy Controls (*n* = 157)	
	Men (*n* = 80)	Women (*n* = 306)	Men (*n* = 42)	Women (*n* = 115)
**Do you consider yourself sexually attractive?**				
Not at all	26.25% (21)	26.14% (80)	-	6.09% (7)
Less than average	12.5% (10)	19.61% (60)	7.14% (3)	12.17% (14)
Average	48.75% (39)	39.22% (120)	42.86% (18)	56.52% (65)
More than average	10% (8)	12.75% (39)	45.24% (19)	13.91% (16)
Very attractive	2.5% (2)	2.28% (7)	4.76% (2)	11.3% (13)

Results are expressed as relative (absolute) frequencies. HS: hidradenitis suppurativa.

**Table 4:** Sexuality in patients with HS in a stable relationship.

Sexuality in Patients in a Stable Relationship	Men (*n* = 54)	Women (*n* = 236)	All (*n* = 290)
**Your partner**			
Helps you overcome the fear of rejection	37% (20)	49.6% (117)	47.2% (137)
Supports you	64.8% (35)	73.3% (173)	71.7% (208)
Helps with lesion dressing in intimate areas	63% (34)	47% (111)	50% (145)
None of the above	9.3% (5)	10.6% (25)	10.3% (30)
**Do you think that HS negatively influences your relationship?**			
0: Not at all	31.5% (17)	28% (66)	28.6% (83)
1: A little	35.2% (19)	44.9% (106)	43.1% (125)
2: Somewhat	18.5% (10)	18.6% (44)	18.6% (54)
3: Very much	14.8% (8)	8.5% (20)	9.7% (28)

Results are expressed as relative (absolute) frequencies. HS: hidradenitis suppurativa.

**Table 5:** Sexuality in single patients with HS.

Sexuality in Single Patients	Men (*n* = 26)	Women (*n* = 70)	All (*n* = 96)
**How do you feel when you meet someone you could have a relationship or sexual relations with?**			
“I feel good, excited”	34.6% (9)	20% (14)	24% (23)
“I am afraid of rejection and the reaction of the other person because of HS”	53.8% (43)	71.4% (50)	66.7% (64)
“I prefer not to meet anyone because of HS”	15.4% (4)	27.1% (19)	24% (23)
“I prefer not to meet anyone for other reasons”	15.4% (4)	5.7% (4)	8.3% (8)
**Do you think that HS negatively influences your chances of having a relationship or sexual relations?**			
0: Not at all	19.2% (5)	5.7% (4)	9.4% (9)
1: A little	19.2% (5)	37.1% (26)	32.3% (31)
2: Somewhat	38.5% (10)	25.7% (18)	29.2% (28)
3: Very much	23.1% (6)	31.4% (22)	29.2% (28)

Results are expressed as relative (absolute) frequencies. HS: hidradenitis suppurativa.

## Supplementary Materials

The following are available online at https://www.mdpi.com/1660-4601/17/23/8808/s1, File S1: Cuestionario Para Pacientes.
