# Supplementary material for: Sexuality in Patients with Hidradenitis Suppurativa: Beliefs, Behaviors and Needs
Source: Int J Environ Res Public Health. 2020 Nov 27;17(23):8808. doi: 10.3390/ijerph17238808 (PMC7730576; doi:10.3390/ijerph17238808)
Supplement: Supplementary file 1 [file ijerph-17-08808-s001.pdf]

CUESTIONARIO PARA PACIENTES

1. CARACTERÍSTICAS GENERALES

Año de nacimiento: _______

Sexo: ☐Mujer ☐Varón

País de residencia: ☐España ☐Otro (por favor, indíquelo): _______

Nivel de estudios: ☐Básico ☐Secundario ☐Superior

¿Cuánto pesa? (indique su peso en Kg): _______

¿Cuánto mide? (indique su altura en cm): _______

Indique si fuma actualmente: ☐Sí ☐No

¿Toma alguna medicación de forma habitual? Por favor, indíquela (por ejemplo, enalapril, simvastatina…): ____

¿Qué edad tenía cuando comenzó la hidradenitis supurativa?: _______

¿Cuántos años hace que recibe atención médica para la hidradenitis supurativa?: _______

¿Cuántos años tardaron en diagnosticarle la hidradenitis supurativa?: _______

En el último mes, ¿en qué zonas de su cuerpo tiene o ha tenido LESIONES ACTIVAS o CICATRICES? Por favor, marque las casillas que correspondan:

	LESIONES ACTIVAS	CICATRICES
Axilas		
Ingles		
Genitales		
Glúteos		
Región mamaria		
Abdomen		
Región perianal		
Cuello		

Piense un momento en la zona de su cuerpo más afectada por la hidradenitis supurativa. ¿A cuál de estas tres imágenes se parece más? Por favor, indíquela (marque solo una respuesta):

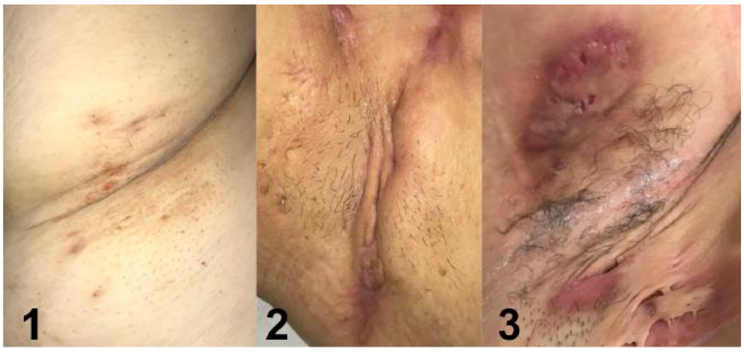

☐1 ☐2 ☐3

En el último mes, ¿cómo diría que es la actividad de su enfermedad? Marque solo una respuesta

☐1 (inactiva)

☐2 (muy leve)

☐3 (leve)

☐4 (moderada)

☐5 (grave)

En el último mes, si 0 es no tener ningún síntoma y 10 la máxima intensidad posible, ¿cuánto DOLOR ha sentido por la hidradenitis supurativa? Marque solo una respuesta

	0	1	2	3	4	5	6	7	8	9	10	
Inexistente	◯	◯	◯	◯	◯	◯	◯	◯	◯	◯	◯	Lo máximo posible

En el último mes, si 0 es no tener ningún síntoma y 10 la máxima intensidad posible, ¿cuánto PICOR ha sentido por la hidradenitis supurativa? Marque solo una respuesta

	0	1	2	3	4	5	6	7	8	9	10	
Inexistente	◯	◯	◯	◯	◯	◯	◯	◯	◯	◯	◯	Lo máximo posible

En el último mes, si 0 es no tener ningún síntoma y 10 la máxima intensidad posible, ¿cuánto MAL OLOR ha tenido por la hidradenitis supurativa? Marque solo una respuesta

	0	1	2	3	4	5	6	7	8	9	10	
Inexistente	◯	◯	◯	◯	◯	◯	◯	◯	◯	◯	◯	Lo máximo posible

En el último mes, si 0 es no tener ningún síntoma y 10 la máxima intensidad posible, ¿cuánta SUPURACIÓN ha tenido por la hidradenitis supurativa? Marque solo una respuesta

	0	1	2	3	4	5	6	7	8	9	10	
Inexistente	◯	◯	◯	◯	◯	◯	◯	◯	◯	◯	◯	Lo máximo posible

2. **DIFICULTADES PERCIBIDAS EN LA ACTIVIDAD SEXUAL**

¿Cómo dificulta la hidradenitis supurativa su vida sexual? Puede marcar más de una respuesta

☐Hace que tenga miedo al rechazo o a la reacción de mi pareja sexual

☐Dificulta mis relaciones sexuales por el dolor

☐Dificulta mis relaciones sexuales por la supuración

☐Dificulta mis relaciones sexuales por el mal olor

☐El tratamiento de la hidradenitis supurativa dificulta mis relaciones sexuales

☐Ninguna de las anteriores

¿Le gustaría compartir sus problemas sexuales con personal sanitario? Puede marcar más de una respuesta

☐No

☐Sí, con mi médico de familia

☐Sí, con mi dermatólogo

☐Sí, con personal de enfermería

☐Sí, con un psicólogo/sexólogo

3. **ATRACTIVO PERCIBIDO**

¿Se considera atractivo/a sexualmente? Marque solo una respuesta

☐Nada

☐Un poco

☐Lo normal

☐Bastante

☐Mucho

4. **ORIENTACIÓN SEXUAL**

¿Por quién siente atracción sexual? Marque solo una respuesta

☐Los hombres

☐Las mujeres

☐Los hombres y las mujeres

¿Tiene pareja estable?

☐Sí (pasa automáticamente al apartado 5)

☐No (pasa automáticamente al apartado 6)

5. SEXUALIDAD EN PACIENTES CON PAREJA ESTABLE

Su pareja estable: (puede marcar más de una respuesta)

☐Te ayuda a vencer el miedo al rechazo

☐Te apoya

☐Te ayuda con las curas en tus zonas íntimas

☐Ninguna de las anteriores

¿Cree que la hidradenitis supurativa influye negativamente sobre su relación de pareja? Marque solo una respuesta

☐Nada

☐Un poco

☐Bastante

☐Mucho

6. SEXUALIDAD EN PACIENTES SOLTEROS

¿Cómo se siente al conocer a alguien con quien podría tener una relación de pareja o relaciones sexuales? Puede marcar más de una respuesta

☐Me siento bien, ilusionado/a

☐Tengo miedo al rechazo y a la reacción de la otra persona por la hidradenitis supurativa

☐Prefiero no conocer a nadie a causa de la hidradenitis supurativa

☐Prefiero no conocer a nadie por otros motivos

¿Cree que la hidradenitis supurativa influye negativamente sobre sus posibilidades de tener una relación de pareja o relaciones sexuales? Marque solo una respuesta

☐Nada

☐Un poco

☐Bastante

☐Mucho

CUESTIONARIO PARA CONTROLES SANOS

Año de nacimiento: _______

Sexo: ☐Mujer ☐Varón

País de residencia: ☐España ☐Otro (por favor, indíquelo): _______

Nivel de estudios: ☐Básico ☐Secundario ☐Superior

¿Cuánto pesa? (indique su peso en Kg): _______

¿Cuánto mide? (indique su altura en cm): _______

Indique si fuma actualmente: ☐Sí ☐No

¿Toma alguna medicación de forma habitual? Por favor, indíquela (por ejemplo, enalapril, simvastatina…): ____

¿Se considera atractivo/a sexualmente? Marque solo una respuesta

☐Nada

☐Un poco

☐Lo normal

☐Bastante

☐Mucho

¿Por quién siente atracción sexual? Marque solo una respuesta

☐Los hombres

☐Las mujeres

☐Los hombres y las mujeres

¿Alguna vez ha sentido miedo al rechazo de su pareja sexual/sentimental?

☐No

☐Sí

En caso de que alguna vez haya sentido miedo al rechazo, indique el/los motivo(s): Puede marcar más de una respuesta (pregunta disponible solamente para personas que respondieran "sí" al ítem anterior):

☐Por mi apariencia física

☐Por inseguridad

☐Por problemas emocionales (como ansiedad, depresión…)

☐Por un problema de salud

☐Por otros motivos

Si tuviera problemas sexuales, ¿le gustaría compartirlos con personal sanitario? Puede marcar más de una respuesta

☐No

☐Sí, con mi médico de familia

☐Sí, con personal de enfermería

☐Sí, con un psicólogo/sexólogo

¿Tiene pareja estable?

☐Sí

☐No

¿Cómo se siente al conocer a alguien con quien podría tener una relación de pareja o relaciones sexuales? (pregunta disponible solamente para personas que respondieran “no” en el ítem anterior)

☐Me siento bien, ilusionado/a

☐Tengo miedo al rechazo y a la reacción de la otra persona por algún motivo

QUESTIONNAIRE FOR PATIENTS

1. BASELINE CHARACTERISTICS

Birth year: _______

Sex: ☐Female ☐Male

Residence country: ☐Spain ☐Abroad (please, indicate): _______

Educational status: ☐Basic ☐Medium ☐Superior

How much do you weigh? (indicate your weight in Kg): _______

How tall are you? (indicate your height in cm): _______

Indicate if you currently smoke: ☐Yes ☐No

Do you take any medication on a regular basis? Please indicate it (e.g. enalapril, simvastatin…): ____

How old were you when hidradenitis suppurativa started? _______

How many years have you been receiving medical attention for hidradenitis suppurativa? _______

How many years did it take for you to be diagnosed with hidradenitis suppurativa? _______

In the last month, in what areas of your body do you have or have you had ACTIVE LESIONS or SCARS? Please check the boxes that apply:

	ACTIVE LESIONS	SCARS
Axilla		
Groin		
Genitals		
Buttocks		
Breast		
Abdomen		
Perianal region		
Neck		

Think for a moment about the area of your body most affected by hidradenitis suppurativa. Which of these three images does it look most like? Please indicate it (check only one answer):

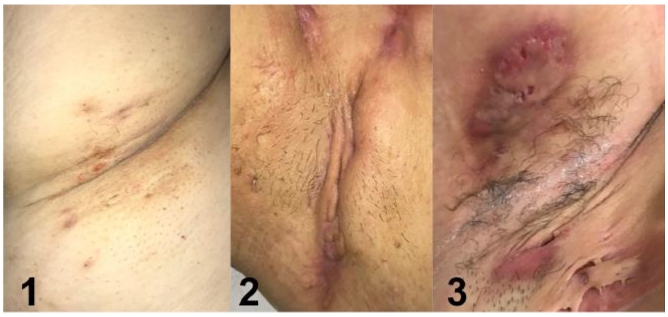

☐1 ☐2 ☐3

In the last month, how would you rate the activity of your disease? Check only one answer:

☐1 (inactive)

☐2 (very low)

☐3 (mild)

☐4 (moderate)

☐5 (severe)

In the last month, if 0 is no symptom and 10 is the maximum intensity possible, how much PAIN have you felt because of hidradenitis suppurativa? Check only one answer

	0	1	2	3	4	5	6	7	8	9	10	
Absent	◯	◯	◯	◯	◯	◯	◯	◯	◯	◯	◯	Maximum possible

In the last month, if 0 is no symptom and 10 is the maximum intensity possible, how much ITCH have you felt because of hidradenitis suppurativa? Check only one answer

	0	1	2	3	4	5	6	7	8	9	10	
Absent	◯	◯	◯	◯	◯	◯	◯	◯	◯	◯	◯	Maximum possible

In the last month, if 0 is no symptom and 10 is the maximum intensity possible, how much BAD ODOR have you felt because of hidradenitis suppurativa? Check only one answer

	0	1	2	3	4	5	6	7	8	9	10	
Absent	◯	◯	◯	◯	◯	◯	◯	◯	◯	◯	◯	Maximum possible

In the last month, if 0 is no symptom and 10 is the maximum intensity possible, how much SUPPURATION have you felt because of hidradenitis suppurativa? Check only one answer

	0	1	2	3	4	5	6	7	8	9	10	
Absent	◯	◯	◯	◯	◯	◯	◯	◯	◯	◯	◯	Maximum possible

2. PERCEIVED DIFFICULTIES IN SEXUAL ACTIVITY

How does hidradenitis suppurativa affect you sexual relations? You can check more than one answer

☐I experience fear of rejection or of the reaction of my sexual partner

☐Pain interferes with my sexual relations

☐Suppuration interferes with my sexual relations

☐Odor interferes with my sexual relations

☐Treatment interferes with my sexual relations

☐None of the above

Would you like to share your sexual problems with healthcare staff? You can check more than one answer

☐No

☐Yes, with my GP

☐Yes, with my dermatologist

☐Yes, with nursing staff

☐Yes, with a psychologist/sexologist

3. PERCEIVED ATTRACTIVENESS

Do you consider yourself sexually attractive? Check only one answer

☐Not at all

☐Less than average

☐Average

☐More than average

☐Very attractive

4. SEXUAL PREFERENCES

Who are you sexually attracted to? Check only one answer

☐Men

☐Women

☐Both men and women

Are you in a stable relationship?

☐Yes (automatically goes to section 5)

☐No (automatically goes to section 6)

5. **SEXUALITY IN PATIENTS WITH HIDRADENITIS SUPPURATIVA IN STABLE RELATIONSHIP**

Your partner: (you can check more than one answer)

☐Helps you overcome the fear of rejection

☐Supports you

☐Helps with lesion dressing in intimate areas

☐None of the above

Do you think that hidradenitis suppurativa negatively influences your relationship? Check only one answer

☐Not at all

☐A little

☐Somewhat

☐Very much

6. **SEXUALITY IN SINGLE PATIENTS WITH HIDRADENITIS SUPPURATIVA**

How do you feel when you meet someone you could have a relationship or sexual relations with? You can check more than one answer

☐I feel good, excited

☐I am afraid of rejection and the reaction of the other person because of hidradenitis suppurativa

☐I prefer not to meet anyone because of hidradenitis suppurativa

☐I prefer not to meet anyone for other reasons

Do you think that HS negatively influences your chances of having a relationship or sexual relations? Check only one answer

☐Not at all

☐A little

☐Somewhat

☐Very much

QUESTIONNAIRE FOR CONTROLS

Birth year: _______

Sex: ☐Female ☐Male

Residence country: ☐Spain ☐Abroad (please, indicate): _______

Educational status: ☐Basic ☐Medium ☐Superior

How much do you weigh? (indicate your weight in Kg): _______

How tall are you? (indicate your height in cm): _______

Indicate if you currently smoke: ☐Yes ☐No

Do you take any medication on a regular basis? Please indicate it (e.g. enalapril, simvastatin…): ____

Do you consider yourself sexually attractive? Check only one answer

☐Not at all

☐Less than average

☐Average

☐More than average

☐Very attractive

Who are you sexually attracted to? Check only one answer

☐Men

☐Women

☐Both men and women

Have you ever felt fear of rejection of your sexual partner?

☐No

☐Yes

If you have ever felt fear of rejection, please indicate the reason(s): You can check more than one answer (question available for people who answered "yes" to the previous item)

☐Because of my physical appearance

☐Because of insecurity

☐Because of emotional problems (such as anxiety, depression…)

☐Because of a health problem

☐For other reasons

If you had sexual problems, would you like to share them with healthcare staff? You can check more than one answer

☐No

☐Yes, with my GP

☐Yes, with nursing staff

☐Yes, with a psychologist/sexologist

Are you in a stable relationship?

☐Yes

☐No

How do you feel when you meet someone you could have a relationship or sexual relations with? (question available for people who answered “no” to the previous item)

☐I feel good, excited

☐I am afraid of rejection and the reaction of the other person for whatever reason
